# No association between joint hypermobility, musculoskeletal pain and neurodevelopmental problems in a school-based sample of 11-year-old children

**DOI:** 10.1192/bjo.2025.10881

**Published:** 2025-11-03

**Authors:** Martin R. Glans, Adyan Aziz, Erik Kindgren, Rajna Knez, Magnus Landgren, Valdemar Landgren

**Affiliations:** Department of Psychiatry, Psychiatry Southwest (Psykiatri Sydväst), Region Stockholm, Huddinge, Sweden; Gillberg Neuropsychiatry Centre, Institute of Neuroscience and Physiology, Sahlgrenska Academy, University of Gothenburg, Sweden; Department of Paediatrics, Skaraborg Hospital, Skövde, Sweden; Department of Psychiatry, Skaraborg Hospital, Skövde, Sweden

**Keywords:** Joint hypermobility, Ehlers–Danlos syndrome, neurodevelopmental disorders, ESSENCE, pain

## Abstract

**Background:**

Adult cohorts with generalised joint hypermobility (GJH) report higher rates of neurodevelopmental problems (NDPs). However, the prevalence of GJH in community-dwelling children and its association with NDPs remains unexplored.

**Aims:**

This study aimed to (a) assess the prevalence of GJH, (b) examine its link to musculoskeletal pain and (c) explore associations with NDPs in 11-year-old Swedish children.

**Method:**

An in-school study was conducted as part of the 4th grade health check-up. It included a structured physical examination using the Beighton score (range 0–9) and a comprehensive neurodevelopmental assessment based on behavioural ratings, maternal interviews, medical records and academic performance.

**Results:**

Of 348 eligible children from eight schools, 223 (64%) participated, with Beighton scores measured in 207 (59%). The median Beighton score was 1 (interquartile range 0–2), with no significant gender differences (Wilcoxon test, *P* = 0.17). A Beighton score of ≥6 approximated the 95th percentile in both sexes. No significant association was found between high Beighton scores and NDPs. Few children with GJH reported weekly pain, indicating a low prevalence of hypermobility spectrum disorders in this age group.

**Conclusions:**

Our findings validate the age-specific Beighton score cut-off and suggest that GJH in children of this age is not linked to NDPs, differing from findings in adults. This may reflect developmental changes during puberty. Additionally, the high prevalence of weekly pain (42%) in the cohort warrants further investigation into its causes and impact.

Recognising and understanding commonly co-occurring health conditions, often referred to as multimorbidity, is crucial for the delivery of targeted and coordinated interventions to affected individuals. Multimorbidity significantly impairs health-related quality of life, particularly for those with physical/mental multimorbidity, who face a disproportionately higher risk.^1^ However, research has mainly focused on adults. Further insights on multimorbidity could shed light on disease progression and the underlying aetiopathogenetics.^2^ Recently there has been growing awareness of the somewhat unexpected association between joint hypermobility and neurodevelopmental problems (NDPs), both believed to emerge predominantly in childhood and adolescence.

## Joint hypermobility and associated conditions

Joint hypermobility describes an increased range of motion in synovial joints, and is termed generalised joint hypermobility (GJH) when it affects multiple joints (typically at least four, depending on age and assessment tool).^3^ GJH is estimated to affect approximately 10–20% of the general population.^4^ However, reported prevalence rates vary widely among studies due to factors including age, gender, race and assessment method.^4^ A large Swedish study from 2004 reported a prevalence of 15% in girls and 6% in boys at the age of 12 years, if the current age-specific recommendations of ≥6/9 on the Beighton score were to be applied.^5^ In Swedish adults, GJH rates were noted at 11% in females and 5% in males.^6,7^ GJH is an umbrella term that does not inherently differentiate between symptomatic and asymptomatic presentations, a distinction that remains debated and subject to evolving definitions. When systemic manifestations such as musculoskeletal issues (e.g. pain, joint instability, dislocations), cutaneous manifestation (e.g. stretchy skin, striae, atrophic scars) and varying degrees of tissue fragility (e.g. recurrent hernias, vascular aneurysms) accompany GJH, it may be part of a hereditary connective tissue disorder (HCTD) such as Ehlers–Danlos Syndrome (EDS), affecting approximately 1 in 5000 individuals.^8^ For symptomatic GJH cases not meeting the diagnostic HCTD criteria, the term generalised hypermobility spectrum disorder (gHSD) is used; gHSD encompasses GJH with related musculoskeletal manifestations, such as recurring or chronic musculoskeletal pain or frequent joint dislocations. Prevalence estimates for gHSD are scarce, ranging from 0.75 to 2.0% in the general population, assuming that about 10% of individuals with GJH experience symptomatic GJH.^9^ The progression of GJH to symptomatic GJH remains uncertain,^10,11^ and studies investigating the relationship between GJH and musculoskeletal pain in children have produced conflicting results.^11–16^ For most types of EDS, responsible genes encoding for collagens and enzymes involved in their biosynthesis, or the maintenance of extracellular matrix homeostasis, have been identified. The underlying mechanisms for GJH, gHSD and hypermobile EDS (hEDS) remain unknown, but are thought to involve a similar pathogenesis.^3,8^ Collagens are abundant in humans, constituting approximately 25% of the body’s total protein content; as a fundamental component of the extracellular matrix, they provide structural support and shape to tissues. Additionally, they interact with cells through various receptor families, thereby regulating their proliferation, migration and differentiation throughout the body.^17^ Consequently, the increased awareness of burdens beyond the core musculoskeletal and cutaneous features of the joint hypermobility spectrum is not surprising. A recent expert consortium on children with gHSD and EDS recognised specific conditions as ‘core comorbidities’ (defined as having evidence from at least cohort studies and a prevalence greater than 10%), including chronic pain, chronic fatigue, functional gastrointestinal disorders, functional bladder disorders, dysautonomia and anxiety. It also identified several ‘emerging comorbidities’ (limited current evidence or probably low frequency), including attention-deficit hyperactivity disorder (ADHD), autism spectrum disorder (ASD) and developmental coordination disorder (DCD).^10^

## Joint hypermobility and psychiatry

With regard to psychiatry, the most robust relationship with joint hypermobility in child cohorts is for ADHD.^18–21^ Additionally, there are varying levels of evidence for a link with ASD,^20,22,23^ DCD^24–28^ and anxiety disorders.^29,30^ In adults, anxiety disorders and ADHD have shown robust associations, with emerging evidence for ASD, mood disorders depression and eating disorders.^6,7,31^ NDPs rarely present with a clear and well-defined clinical picture. The ‘early symptomatic syndromes eliciting of neurodevelopmental clinical examinations’ (ESSENCE) concept recognises that early symptoms and signs of developmental deviations are non-specific, prompt further clinical evaluations and eventually align as neurodevelopmental disorders (NDDs), where ‘comorbidity’ across NDDs (e.g. ADHD and ASD) is the rule rather than the exception.^32^ Because ESSENCE problems are believed to be prevalent within the joint hypermobility spectrum,^24,33^ GJH may serve as an additional clinical clue during health check-ups. To our knowledge, no studies have explored the link between GJH and ESSENCE in general population screening settings. Moreover, further exploration of this presumed association could provide valuable insights into disease mechanisms. Some researchers suggest that collagens, or pleiotropy in collagen-related genes, play a simultaneous role in the development and functioning of the central nervous system, in addition to their involvement in joint mobility.^34^

In summary, joint hypermobility is associated with a broad range of NDPs. Further research is needed to understand how collagen abnormalities may relate to disruptions in central nervous system development and function. Moreover, enhancing our understanding of GJH, gHSD and musculoskeletal pain in children can contribute to health initiatives and scientific progress.

## Aims

This study aimed to (a) determine GJH and gHSD prevalence, (b) assess the correlation between GJH and musculoskeletal pain and (c) investigate potential associations between GJH and gHSD, with a comprehensive array of NDPs at different symptom levels in 11-year-old Swedish children from the general population

## Method

### Participants and procedure

**Fig. 1:**
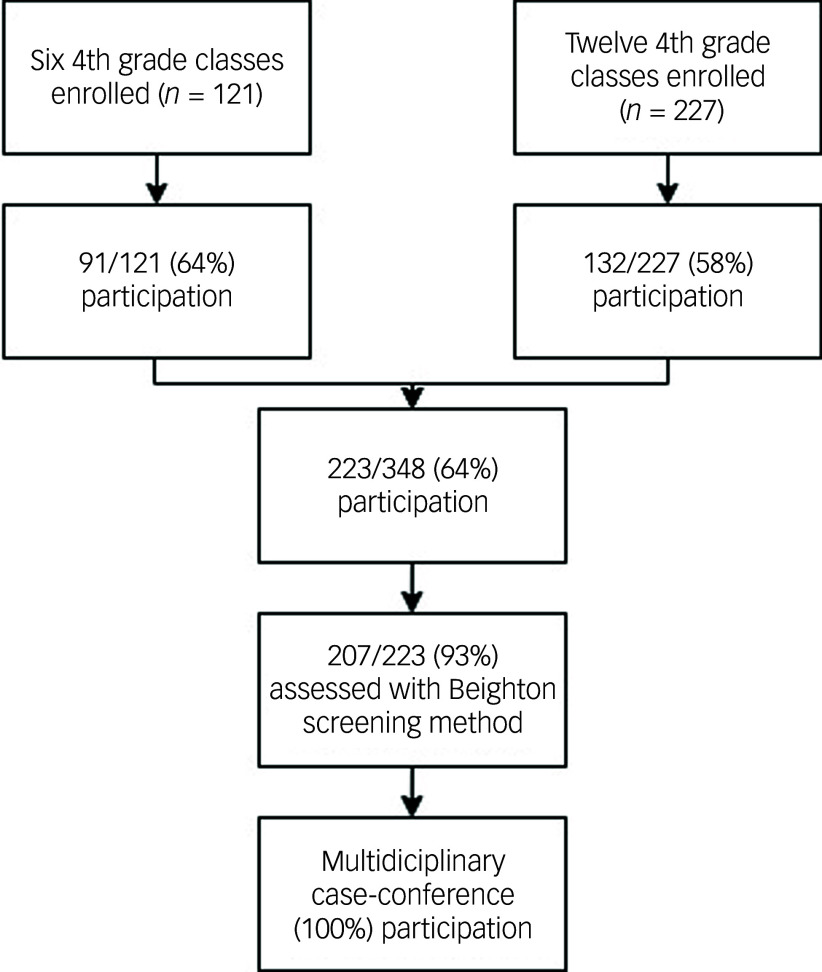
Participant flow diagram.

This cross-sectional study took place in eight schools – six public and two private – in western Sweden from 2018 and 2020, and has been reported in detail previously.^35^ This report is part of a broad research initiative covering pupils’ basic academic skills, general health and neurodevelopment, and possible associations with important background factors. Schools were selected based on local school principals’ readiness to participate in studies of children’s health. Caregivers of all pupils attending 4th grade were invited following an oral and written presentation of the study. At each school, one or two academic years of children were recruited. Participation constituted an add-on to the existing regular health check-up conducted in the 4th grade. Data sources included medical health records, psychological assessment, physician-led physical assessment, maternal interviews, teacher and parent rating scales and national tests from the 3rd grade (Fig. 1). Following data collection, a case conference, attended by a psychiatrist, psychologist and a paediatric neurologist, was held to summarise the clinical information. Where sufficient data were available, medical conditions and psychiatric and neuropsychiatric problem areas were assigned. A detailed description of this procedure is reported elsewhere.^35^

### Measures

#### Exposures

##### GJH

GJH was measured and rated according to the Beighton score^36^ (range 0–9), as part of a structured physical examination that included a neuromotor and dysmorphological assessment. Assessors received training in the structured neurodevelopmental examination from a paediatric neurologist (M.L.), and the protocol encompassed written instructions detailing the criteria for the Beighton score. Joint hypermobility was assessed by four clinicians. A goniometer was not used. Participants did not warm up prior to the examination. The recommended age-adapted cut-off score of ≥6/9 on the Beighton score was used as the criterion for GJH.^10^ To facilitate comparisons with prior research employing lower thresholds, and to address potential dimensional considerations, supplementary analyses were conducted using thresholds of ≥5/9 and ≥4/9, and also with total Beighton score as a continuous variable.

##### HSDs

A proxy variable for gHSD was defined for individuals displaying GJH along with self-reported musculoskeletal pain (described below). To meet the criteria for gHSD, participants were required to exhibit GJH (based on the employed Beighton score cut-off), along with musculoskeletal pain reported weekly.

### Outcomes

#### Musculoskeletal pain

As a component of the systematic physical examination, participants were interviewed using questions that specifically addressed pain in the legs, arms and joints. We ensured that participants understood the distinctions and provided clarification through visual feedback. The participants were provided with three choices for answering. The first option, ‘Often’, referred to pain that occurred more than once a week and was assigned a value of 2 points. The second option, ‘Sometimes’, referred to pain that occurred a few times per month and was assigned a value of 1 point. The third option, ‘Never’, referred to pain that either never or rarely occurred and was assigned a value of 0 points. This allowed for the calculation of a ‘pain score’ ranging from 0 to 6. Analyses of pain encompassed assessment of the relationship between GJH and any reported weekly pain, as well as examination of the correlation between total pain score and total Beighton score. Additionally, responses were utilised to establish the aforementioned proxy variable for gHSD. In this case, the response ‘often’ in any location (pain, leg or arm) was deemed to indicate significant musculoskeletal pain.

#### NDPs

In the case conference summarising clinical information, an emphasis was placed on symptoms of NDDs. For this purpose we scrutinised all available data as reported previously in detail.^35^ The shared polygenetic origins of NDDs, and the frequent co-occurrence of symptoms from multiple diagnostic groups (e.g. autistic traits are common among patients with attention-deficit/hyperactivity disorder), are well established and warrant viewing as dimensional traits rather than discrete entities.^37^ We therefore chose to incorporate a global assessment of the NDPs (i.e. NDD symptoms irrespective of diagnostic status) displayed by each participant. This allowed us not only to categorise individuals who might fall below a clinical diagnostic threshold, but also to take into account the total burden of symptoms for each participant.

For each participant, the overall severity of symptoms/degree of functional impairment from NDPs was rated using the ordinal scale Clinical Global Impression-Severity instrument (CGI-S, range 1–7).^38^ The CGI rating thus took into account multiple functional areas (e.g. cognition, hyperactivity/inattention, autistic traits, coordination problems) and rated the symptoms and impairment irrespective of whether a diagnostic cut-off was met or not. In a clinical setting based on our experience, participants with CGI 1–3 would not be referred for regular healthcare, while those with CGI 4–7 would probably indicate symptoms/impairments warranting clinical attention and likely diagnosis.

For analysis, we chose the total score and hyperactivity subscore from the Strength and Difficulties Questionnaire (SDQ).^39^ This choice was made due to the broad validation of SDQ as a measure of NDDs in research.^40^ Additionally, SDQ has been utilised in prior studies on ADHD, a neurodevelopmental condition linked to GJH.^6^ We also considered SDQ as a complement to our own global measure.

### Statistics

We used percentages for displaying proportions in descriptive statistics. Continuous variables were reported with the mean and standard deviation. In instances of small groups and non-normal distributions following visual inspection, median and interquartile range were reported. Anthropometric variables were converted to *z*-scores that denote the distance of the raw score from that of the reference mean, and were handled as continuous variables.

Prevalence rates and 95% confidence intervals for GJH and HSD were estimated according to the Clopper–Pearson exact method. We conducted separate analyses by gender, and at three different cut-offs on the Beighton score. Because sample size was not contingent upon the current objective, we examined the statistical power for the present hypotheses. Applying an alpha of 0.05 and a beta of 0.8, the hypothesis comparing GJH at the 95th percentile with those below, regarding binary variables, suggested power to detect only large effect sizes (∼0.8). To mitigate type II errors (false negatives), we also designed analyses for continuous variables because the sample size provided sufficient power to identify even minor effects for these (∼0.2).

We split the cohort into high- and low-score groups regarding Beighton score, with a cut-off of ≥6. The same was applied to gHSD, which was defined as weekly pain in combination with Beighton score ≥6. We used Fisher’s test to compare groups for binary variables, and the Mann–Whitney *U*-test to compare continuous variables. Spearman’s rank correlation analysis was used to investigate relationships between Beighton score and specific factors of interest that could be analysed as dimensional variables (pain score, SDQ score and CGI ratings of NDPs).

As sensitivity analyses we analysed high- and low-score groups, and the gHSD definition with additional cut-offs (≥4, ≥5). We also investigated associations between Beighton score and CGI-S, the measure of global burden of NDPs, while adjusting for the impacts of gender and body mass index (BMI). This was performed with Beighton score, gender and BMI as predictor, and CGI-S as outcome, in a linear regression. Analyses were conducted in R version 4.2.3 for Windows, (Posit Software, PBC, Boston, MA, USA; http://www.posit.co/) using the packages binom, pwr and glm. The manuscript was prepared using Microsoft Word, version 16 (2021; Microsoft Corporation, Redmond, WA, USA; https://www.microsoft.com/microsoft-365/word running on macOS 11 Big Sur (2020; Apple Inc., Cupertino, CA, USA; https://www.apple.com/macos/big-sur).

## Results

### Characteristics

Of 348 eligible students, 223 consented to participation and 207 had been assessed by a physician and could be included in the study (Fig. 1). The participants exhibited slightly greater height and weight compared with the reference values, with boys showing a somewhat greater difference (Table 1).

**Table 1:** Participant characteristics

Chracteristics	Overall	Female	Male
*n*	207	89	118
Age, mean (s.d.)	110 (0.3)	110 (0.4)	11.0 (0.3)
Anthropometry, mean (s.d.)			
Length, cm	147.0 (7.4)	146.1 (7.3)	147.7 (7.3)
Weight, kg	41.1 (9.2)	40.2 (9.5)	41.8 (8.9)
BMI	18.9 (3.3)	18.7 (3.5)	19.0 (3.1)
Anthropometry, mean *z*-score (s.d.)			
Length	0.34 (1.2)	0.21 (1.1)	0.45 (1.2)
Weight	0.75 (1.6)	0.53 (1.7)	0.92 (1.5)
BMI	0.89 (1.5)	0.67 (1.5)	1.05 (1.6)

BMI, body mass index.

#### Prevalence rates of GJH and gHSD

**Fig. 2:**
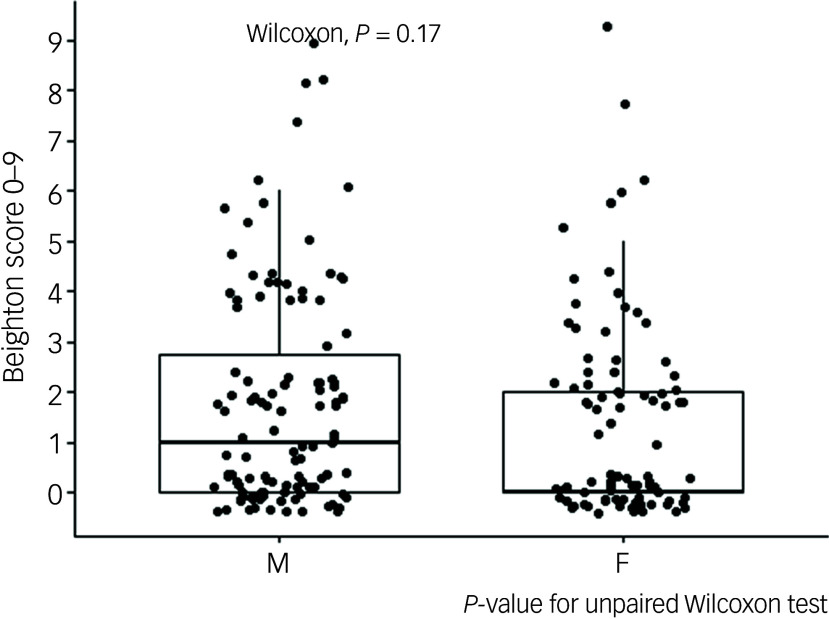
Beighton score stratified by gender. M, male; F, female.

Table 2 presents the distribution and cumulative percentage of Beighton score, along with gender-stratified rates of GJH and gHSD based on various cut-off levels on the Beighton score. Overall, the median Beighton score was 1 (interquartile range 0–2) and did not differ significantly between genders (Wilcoxon test *P* = 0.17; Fig. 2). With the recommended cut-off of ≥6/9 on the Beighton score, 1 participant met the criteria for gHSD. Lowering the cut-off to ≥5/9 and ≥4/9 resulted in 2 and 6 individuals meeting the gHSD criteria, respectively.

**Table 2:** Distribution and cumulative percentage of joint hypermobility

Beighton score	Overall, *n* (%)	Cumulative, %	Girls, *n* (%)	Cumulative, %	Boys, *n* (%)	Cumulative, %
0	96 (46)	46	47 (53)	53	49 (42)	42
1	15 (7)	54	3 (3)	56	12 (10)	52
2	47 (23)	76	20 (23)	79	27 (23)	75
3	9 (4)	81	7 (8)	87	2 (2)	76
4	23 (11)	92	6 (7)	93	17 (14)	91
5	4 (2)	94	1 (1)	94	3 (3)	93
6	7 (3)	97	3 (3)	98	4 (3)	97
7	1 (1)	98	0 (0)	98	1 (1)	97
8	3 (1)	99	1 (1)	99	2 (2)	99
9	2 (1)	100	1 (1)	100	1 (1)	100
Generalised joint hypermobility prevalence^a^						
	Overall, %	95% CI	Girls, %	95% CI	Boys, %	95% CI
Beighton score ≥4	19.3	14.2–25.4	13.5	7.2–22.4	23.7	16.4–32.4
Beighton score ≥5	8.2	4.9–12.8	6.7	2.5–14.1	9.3	4.7–16.1
Beighton score ≥6	6.3	3.4–10.5	5.6	1.8–12.6	6.8	3.0–12.9
Generalised hypermobility spectrum disorder^b^						
Pain + Beighton score ≥4	3.4	1.4–6.8	2.2	0.3–7.9	4.2	1.4–9.6
Pain + Beighton score ≥5	1.0	0.1–3.4	1.1	0–6.1	0.8	0–4.6
Pain + Beighton score ≥6	0.5	0–2.7	1.1	0–6.1	0	0–3.1

a . Generalised joint hypermobility according to different cut-off scores in the Beighton scoring system.

b . Generalised hypermobility spectrum disorder was operationalised as pain (complaints of weekly pain in arms, legs or joints) combined with the specified cut-off in the Beighton scoring system.

### Joint hypermobility and musculoskeletal pain burden

**Fig. 3:**
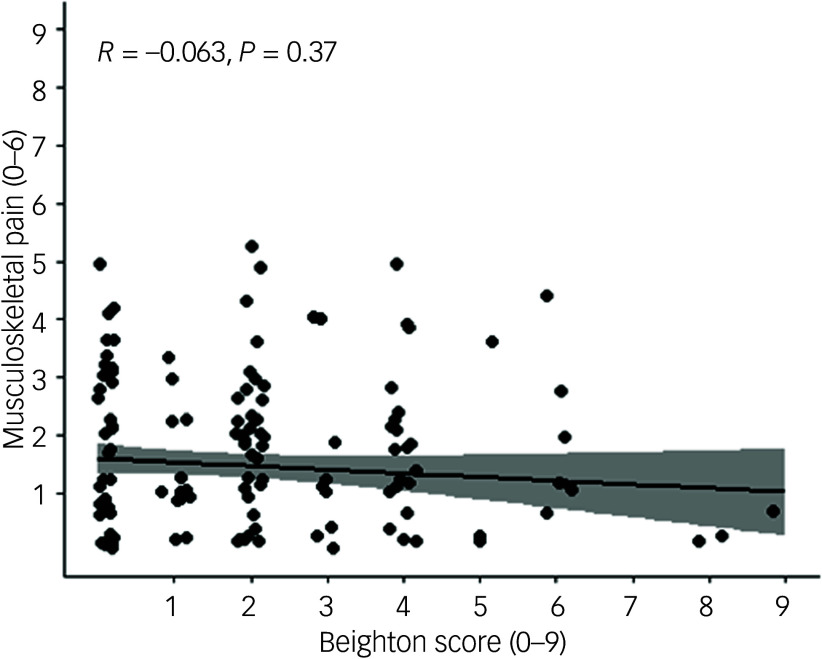
Spearman’s rank correlation between Beighton score and musculoskeletal pain.

Prevalence rates of self-reported weekly pain are reported in Table 3. No significant difference was observed between those with and without GJH. The proportion endorsing weekly pain was insignificantly higher in girls (*n* = 42, 48%) than in boys (*n* = 43, 37%; Fisher’s test *P* = 0.15). There was no significant correlation between total Beighton score and musculoskeletal pain burden (Fig. 3).

**Table 3:** Low- compared with high-scoring participants at Beighton score cut-off ≥6 for generalised joint hypermobility

Participant data	Overall	Non-GJH	GJH	*P* ^b^
*N*	207	194	13	
Males, *n* (%)	118 (57)	110 (57)	8 (62)	0.96
Any weekly pain, *n* (%)	85 (42)	79 (41)	6 (46)	0.95
Neurodevelopmental measures				
Strength and Difficulties Questionnaire^a^				
Total score, mean (s.d.)				
Parent-rated	8.8 (6.9)	8.8 (7.0)	8.8 (4.3)	0.99
Teacher-rated	6.7 (6.1)	6.6 (6.1)	8.2 (5.9)	0.35
Hyperactivity subscale, mean (s.d.)				
Parent-rated	3.4 (2.9)	3.4 (3.0)	2.8 (1.9)	0.52
Teacher-rated	3.1 (3.1)	3.0 (3.1)	4.0 (3.9)	0.29
ESSENCE-Q score, mean (s.d.)	3.9 (5.5)	3.8 (5.4)	5.6 (7.5)	0.36
Non-verbal IQ, mean (s.d.)	107.8 (9.7)	107.6 (9.9)	111.0 (6.5)	0.32
IQ <85, *n* (%)	5 (2.4)	5 (2.6)	0 (0)	1.00
CGI severity of NDPs, mean (s.d.)	2.9 (1.4)	2.9 (1.4)	2.2 (1.0)	0.88
Any degree of NDP (CGI ≥2), *n* (%)	159 (77)	194 (76)	12 (92)	0.30
Clinical-level NDPs (CGI ≥4), *n* (%)	79 (38)	75 (39)	4 (31)	0.79
Symptomatic problem areas, *n* (%)				
Externalising	45 (22)	43 (22)	2 (15)	0.82
Speech, language, learning	10 (5)	9 (5)	1 (8)	1.00
Autism spectrum	12 (6)	12 (6)	0 (0)	0.76
Developmental coordination	21 (10)	20 (10)	1 (8)	1.00

CGI, Clinical Global Impression (range 1–7); ESSENCE-Q, early symptomatic syndromes eliciting of neurodevelopmental clinical examinations (ESSENCE) questionnaire (range 0–24); GJH, generalised joint hypermobility; NDPs, neurodevelopmental problems.

a . The Strength and Difficulties Questionnaire ranges from 0 to 40 in total score and 0–10 in the hyperactivity subscale.

b . *P*-values for Fisher’s exact test and Wilcoxon signed-rank test for binary and continuous variables, respectively.

### Joint hypermobility and NDPs

#### GJH

Participant characteristics stratified as non-GJH or GJH according to Beighton score ≥6 are shown in Table 3. There were no differences between the groups regarding gender and internalising symptoms or occurrence of weekly pain. No difference in any neurodevelopmental measures was observed. Adjusting the cut-off to either ≥5 (*n* = 17 scoring ≥5) or ≥4 (*n* = 40 scoring ≥4) showed no significant differences between the groups (data not shown).

**Fig. 4:**
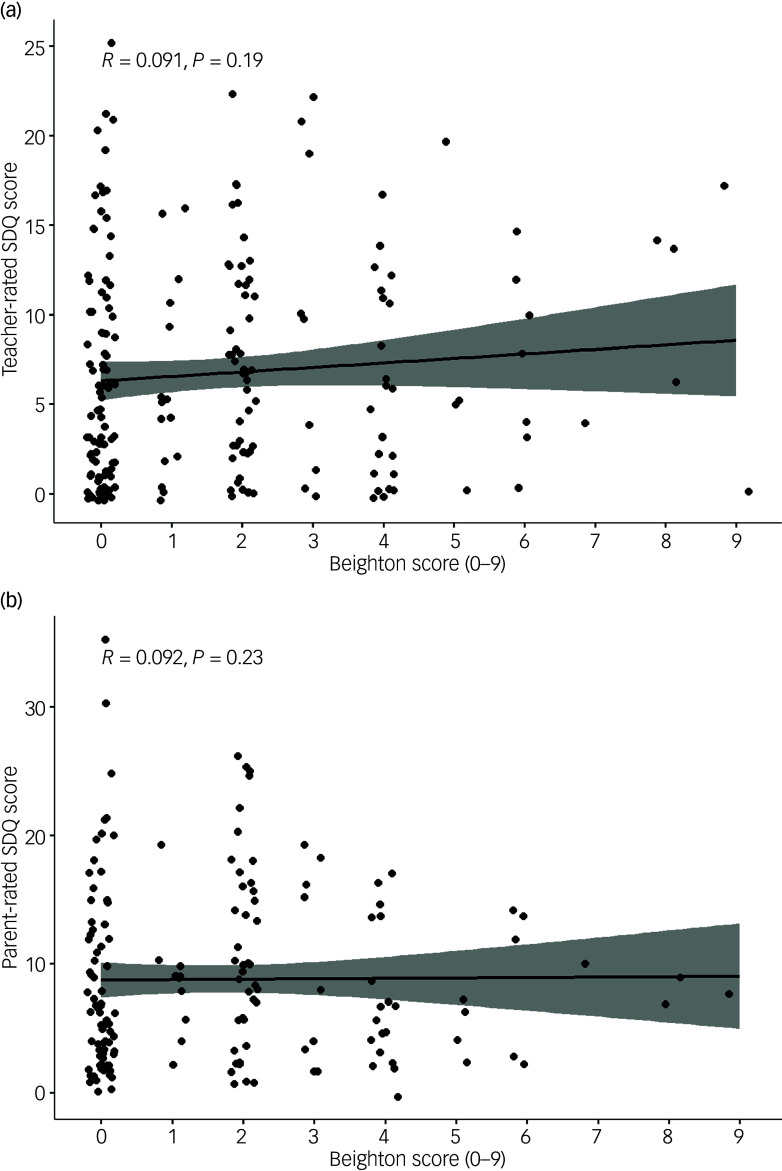
Spearman’s rank correlation between Beighton scores and Strengths and Difficulties Questionnaire (SDQ) scores. (a) Teacher-rated. (b) Parent-rated.

To further explore potential GJH associations of a dimensional nature, we analysed the correlation between total Beighton and total SDQ-scores, but no significant correlation was found (Fig. 4).

To examine potential confounding influences from gender and BMI on the association of GJH and NDPs, we performed a sensitivity analysis; NDP, as rated by CGI-S, was selected as the outcome, and GJH adjusted for gender and BMI as the predictors. No significant association was found for either GJH (estimate 0.04, *P* = 0.32) or gender (estimate 0.19, *P* = 0.31), but a significant association of BMI with NDPs was noted (estimate 0.18, *P* = 0.003). However, taken together, the variance explained by the model was not practically relevant (*R* ^2^ = 0.05, 95% CI: 0–0.11, *P* = 0.01).

#### HSDs

To investigate the potential impact of symptomatic GJH, we operationalised gHSD as the presence of GJH plus weekly musculoskeletal pain. With the recommended cut-off of ≥6/9 on the Beighton score, 1 participant met the criteria for gHSD. Lowering the cut-off to ≥5/9 and ≥4/9 resulted in 2 and 6 individuals meeting the gHSD criteria, respectively; of these 6, 2 had a CGI rating of 4, indicating clinically relevant NDPs. The limited number of individuals meeting the criteria for the gHSD variable prevented meaningful analyses of the association between gHSD and NDPs.

## Discussion

To our knowledge, this study is the first to explore potential associations between joint hypermobility and a comprehensive array of ESSENCE/NDPs in a community sample of middle school-aged children. Employing a cross-sectional design, we measured GJH through physical examination and conducted a thorough evaluation of both subclinical and clinical NDPs by a multidisciplinary team. Our analyses revealed no significant links between GJH and any level of NDP, suggesting that individuals with GJH at this age generally follow typical developmental patterns, thus cautioning against using GJH as a biomarker for NDPs in this age group.

In our cohort, the prevalence rate of GJH was 5.6% for girls and 6.8% for boys. These rates align with the notion that GJH should be assigned to only the most extreme end of the population, and validate the age-dependent recommended cut-off point of ≥6/9 on the Beighton score. We found no association between GJH and musculoskeletal pain. Signs of HSD were uncommon in our sample, affirming its impact on only a subset of individuals and typical emergence later in life. Nevertheless, a significant 42% of the total sample experienced weekly pain, meriting further investigation into its occurrence and impact.

### Prevalence rates of GJH and gHSD

Data on prevalence rates of GJH and gHSD are significant for research, public health policies and raising public awareness. In our study cohort, GJH was identified in 5.6% (95% CI: 1.8–12.6) of girls and 6.8% (95% CI: 3.0–12.9) of boys. Comparing these findings with a 2004 Swedish study involving 12-year-old children, the rates observed among boys were similar but notably lower than those among girls. In the 2004 study, 15% of the girls would have met the criteria for GJH if the current recommended cut-off of ≥6/9 had been applied.^5^ Discrepancies between the two studies may be attributed to hormonal changes and the onset of puberty, probably more pronounced in the 2004 study because participants were 1 year older than in our cohort. These factors are believed to influence joint hypermobility, with an expected increase in prevalence with age among girls and a decrease among boys.^5,41^ Unfortunately, we did not collect data on puberty or biological maturity, which prevents further analysis in this regard. Many researchers and clinicians argue that GJH should signify an anomaly, advocating for setting the cut-off value on the Beighton score to 2 standard deviations above the mean, or within the top 5%, for each age, gender and race category.^5,10,42^ The prevalence rates found in our study validate the use of a cut-off of ≥6/9 on the Beighton score, while also confirming a similar prevalence of GJH between genders in this age group.

GJH, combined with self-reported weekly pain, was used as a proxy variable for gHSD, deviating from the standardised gHSD assessment. These analyses are thus preliminary. With the ≥6/9 cut-off for GJH on the Beighton score, only 1 participant of 207 (0.5%) met the classification for gHSD. Lowering the cut-off to ≥5/9 and ≥4/9 resulted in 2 and 3 individuals meeting the gHSD criteria, respectively. Limited data exist on gHSD prevalence and progression, but the clinical progression of hEDS is believed to unfold in three phases: the hypermobility phase, characterised by hypermobility and frequent joint sprains/strains; the pain phase, marked by widespread chronic pain; and the stiffness phase, with a notable decline in hypermobility.^43^ This pattern may extend to gHSD, supported by the limited number of participants in our cohort manifesting coexisting GJH and pain.

### Joint hypermobility and musculoskeletal pain

Although not within the scope of our primary investigation, the significant prevalence of weekly pain observed in our cohort deserves mention. In this cohort, 42% of the children experienced weekly musculoskeletal pain, with no discernible difference between genders. This exceeds the figure reported in a nationwide Swedish study from 2007, where only 5% of girls and 9% of boys reported weekly musculoskeletal pain.^44^ However, studies on childhood pain yield varied results, with a Norwegian study reporting frequent bodily pain in 30% of children in this age group.^45^ Discrepancies may arise from variations in assessment methods, criteria for defining pain or characteristics of the study population. Musculoskeletal pain in children and adolescents correlates with impaired health-related quality of life,^46^ while also predicting health and social difficulties in later life.^47^ In the present study we did not assess the potential negative impacts of pain. Nevertheless, the significant occurrence observed in our cohort warrants further investigation to deepen our understanding and enable effective detection and prevention.

The analyses on GJH and musculoskeletal pain revealed no significant differences in pain experience between individuals with and without GJH. Additionally, when treating pain and joint hypermobility as dimensional variables, no correlation was observed. These findings align with a systematic review and meta-analysis conducted in 2012, encompassing 15 cross-sectional studies. This meta-analysis revealed no significant correlation between GJH and musculoskeletal pain within Caucasian populations but indicated a link in African Asian populations.^13^ Two subsequent cross-sectional studies also predominantly reported no associations.^11,14^ However, an Australian study noted a relationship for boys when employing the ≥6/9 cut-off on the Beighton score.^14^ While cross-sectional studies have limitations in detecting progression of pain, longitudinal studies provide some support for GJH in childhood as a risk factor for developing musculoskeletal pain in adolescence.^12,15,16^ Joint instability is often cited as a contributor to musculoskeletal pain in GJH,^10^ while central nervous system hypersensitisation is implicated in chronic widespread pain.^12^ Consequently, the aforementioned prospective studies could infer that any potential effects of GJH on joint instability or perceived pain emerge later in life. In conclusion, our study supports the growing evidence of no relationship between GJH and musculoskeletal pain in prepubertal children. However, prospective studies are needed to explore potential changes over time and their relation to biological maturation.

### GJH and NDPs

The present study revealed no significant associations between joint hypermobility and any type of NDP at any level. We evaluated the relationship for two basic variables, GJH and HSD, with a broad spectrum of measures of NDPs/ESSENCE and without confining ourselves to the diagnostic boundaries of DSM-5. This approach offers advantages, given the significant symptom overlap between NDDs and their presumed dimensional nature.^37^ Unlike specific genetic syndromes, GJH probably displays considerable variability among individuals in terms of the underlying mechanisms and associated characteristics. Therefore, it is intriguing that numerous studies have identified robust associations between GJH and various extra-articular conditions. Of note, these associations span the entire joint hypermobility symptom spectrum (i.e. GJH/HSD/EDS), not merely confined to recognised genetic syndromes such as classical EDS or Marfan syndrome. Regarding NDPs on the other hand, it is unknown whether the presumed associations extend across the clinical and non-clinical continuum, because previous studies have predominantly focused on clinical cohorts. To our knowledge, only one study has evaluated the link between joint hypermobility and subclinical NDPs. This study, with a large sample (*n* = 1039) of adults from the general population, found no association between GJH and signs of ADHD, ASD or DCD.^48^

Our results from analysis of subclinical NDPs align with the aforementioned observations in adults.^48^ It is plausible that mechanisms underlying subclinical neurodevelopmental phenotypes are more heterogeneous than those driving more severe pathology, thus diluting the hypothesised influence of collagen abnormality.

However, when assessing more pronounced NDPs, our results deviate from those of previous studies. The relatively low prevalence of GJH (approximately 5%) poses a risk of type 2 errors, particularly in analyses of variables involving fewer participants such as those at higher symptom severity. Nevertheless, it is conceivable that the presumed link between joint hypermobility and NDPs does not apply to children of this age. Recognising the shift within the hypermobile cohort around puberty, marked by a notable influx of females and outflow of males, may be pivotal. Post-pubertal cohorts encompass a notably distinct group of individuals compared with those in prepubertal cohorts. While no prospective studies are available, a Swedish cross-sectional study on hEDS individuals showed an increased ADHD prevalence with age: 35% in 15- to 16-year-olds and 46% in 17- to 18-year-olds, compared with 23% across ages 6–18 years.^21^ Whether this reflects increased evaluation time and awareness of adolescent ADHD, or signals a phenotype shift within the hypermobile cohort around puberty, remains uncertain. There is also a possibility that a hitherto uncharacterised factor (e.g. environmental triggers, personality or lifestyle factors) mediates an association of NDPs and GJH in adulthood. Prospective studies are needed to investigate this matter further. Moreover, because puberty affects hypermobility differently in boys and girls, future studies should consider performing gender-stratified analyses.

### Limitations

This study has several strengths. GJH assessment in all study participants by physical examination ensures high diagnostic accuracy. We explored a wide array of NDPs, extending beyond DSM diagnostic boundaries. This enhanced sensitivity in detecting atypical development, contributing to a comprehensive understanding of these frequently overlapping conditions. Our multidisciplinary team, including psychiatrists, psychologists and paediatric neurologists, enhances reliability and validity. Analysis of NDPs across various symptom levels makes this study unique because it incorporates dimensional aspects of the hypothesised associations. Recruitment during routine school health check-ups facilitated accessibility, inclusivity and data collection efficiency, reducing selection bias.

Our findings should also be considered in light of limitations. No sample size estimation was performed, and assessing variables with low prevalence increases the risk of type 2 errors. The study’s cross-sectional design hinders drawing conclusions about temporal associations. The lack of information about biological maturity or puberty impedes our ability to evaluate potential influences. In addition, joint hypermobility was assessed by multiple clinicians without formal evaluation of interrater reliability, which may have introduced variability in scoring. The attrition rate was substantial and, as previously noted, we interpret the elevated prevalence of NDPs in our cohort as being due to a selection of schools with significant socioeconomic disadvantage, along with a bias towards study participation of families of children with unmet needs who are pursuing an evaluation for NDPs.^35^ Consequently, the sample may have had a higher proportion of NDPs than would be expected in the general population. While this may not directly hinder the capacity to discover genuine associations, it needs to be factored in. The Beighton score primarily focuses on upper limb hypermobility, potentially overlooking hypermobility in joints not included in the Beighton protocol. Despite its limitations, the Beighton score remains the standard assessment method for GJH in both clinical practice and research studies. Lastly, the use of a proxy variable for gHSD and an unvalidated pain score renders these analyses preliminary.

In conclusion, our study on 11-year-old Swedish children showed GJH prevalence rates of 5.6% for girls and 6.8% for boys. These rates align with the notion of capturing 5% of the population, thereby corroborating the current age-dependent recommendations for GJH in the Beighton score.

There was no association between joint hypermobility and musculoskeletal pain, adding to the growing body of evidence that disputes such a link in this age group. Furthermore, our study found that indicators of gHSD were infrequent, confirming that it affects a certain group of individuals with GJH and usually develops at a later stage in life. However, a substantial 42% of the total sample experienced weekly musculoskeletal pain. This significant occurrence merits further investigation to explore potential negative impacts and facilitate detection and prevention.

Finally, our study found no association between joint hypermobility and NDPs, contrasting with previous studies. Based on the age of our study participants, we hypothesise that the absence of such an association could be attributed to significant changes in hypermobility that occur during puberty. Our findings are noteworthy because they suggest that individuals with GJH at this age generally follow typical development trajectories, cautioning against the use of GJH as a biomarker for NDPs in this age group.

## Data Availability

The data that support the findings of this study are available from the corresponding author, M.R.G., upon reasonable request.
